# Methyl 3,4-Dihydroxybenzoate Induces Neural Stem Cells to Differentiate Into Cholinergic Neurons *in vitro*

**DOI:** 10.3389/fncel.2018.00478

**Published:** 2018-12-07

**Authors:** Jun-Ping Pan, Yang Hu, Jia-Hui Wang, Yi-Rong Xin, Jun-Xing Jiang, Ke-Qi Chen, Cheng-You Yang, Qin Gao, Fei Xiao, Li Yan, Huan-Min Luo

**Affiliations:** ^1^Department of Pharmacology, College of Basic Medicine, Jinan University, Guangzhou, China; ^2^Department of Neurosurgery, Guangzhou Women and Children’s Medical Center, Guangzhou, China; ^3^Institute of Brain Sciences, Jinan University, Guangzhou, China; ^4^Department of Neurosurgery, The First Affiliated Hospital of Jinan University, Guangzhou, China; ^5^Guangzhou Quality R&D Center of Traditional Chinese Medicine, Guangdong Key Laboratory of Plant Resources, School of Life Sciences, Sun Yat-sen University, Guangzhou, China

**Keywords:** methyl 3, 4-dihydroxybenzoate, neural stem cells, differentiate, cholinergic neurons, GSK3β, cell cycle, *Isl1*

## Abstract

Neural stem cells (NSCs) have been shown as a potential source for replacing degenerated neurons in neurodegenerative diseases. However, the therapeutic potential of these cells is limited by the lack of effective methodologies for controlling their differentiation. Inducing endogenous pools of NSCs by small molecule can be considered as a potential approach of generating the desired cell types in large numbers. Here, we reported the characterization of a small molecule (Methyl 3,4-dihydroxybenzoate; MDHB) that selectively induces hippocampal NSCs to differentiate into cholinergic motor neurons which expressed synapsin 1 (SYN1) and postsynaptic density protein 95 (PSD-95). Studies on the mechanisms revealed that MDHB induced the hippocampal NSCs differentiation into cholinergic motor neurons by inhibiting AKT phosphorylation and activating autophosphorylation of GSK3β at tyrosine 216. Furthermore, we found that MDHB enhanced β-catenin degradation and abolished its entering into the nucleus. Collectively, this report provides the strong evidence that MDHB promotes NSCs differentiation into cholinergic motor neurons by enhancing gene *Isl1* expression and inhibiting cell cycle progression. It may provide a basis for pharmacological effects of MDHB directed on NSCs.

## Introduction

Neural stem cells (NSCs) have the ability of self-renewal and to differentiate into multiple specialized neural cell types, such as neurons, astrocytes and oligodendrocytes, thus serving as the common source of these fundamental components of the CNS (Gage, 2000). Consequently, NSCs can furnish an unlimited source of cells for cell transplantation therapy to supplement degenerating cells (Ager et al., 2015; Reidling et al., 2018). Facing this potential, some defects, such as the uncontrollability of stem cell differentiation pathway and immune rejection for stem cell therapy, can be surmounted. At present, NSCs have been proved to exist not only in the embryonic mammal nervous system, but also in the nervous system of most adult mammals (Goncalves et al., 2016). In adulthood, the main neurogenic niches are the sub-granular zone (SGZ) of hippocampal dentate gyrus (DG) as well as the sub-ventricular zone (SVZ), both of which continuously generate newborn neurons with potential functions their contribution to behavior, and their relevance to disease (Zhao et al., 2008).

Glycogen synthase kinase 3s are serine/threonine kinases in receptor tyrosine kinase. Wnt/Frizzled signaling pathway is originally confirmed as important regulatory enzymes in glucose metabolism (Hur and Zhou, 2010; Mussmann et al., 2014). There are two subtypes(GSK3α and GSK3β) encoded by different genes, which are overall 85% homologous to each other, with 95% identity in the kinase domains. Kim et al. (2009) showed that the phosphorylation levels of β-catenin, target of GSK3β, are increased at later stages in development when stem cell proliferation is declining and cholinergic neuronal differentiation predominates (Ming and Song, 2009). Degradation of β-catenin by activation of GSK3β will inhibit cell proliferation and increase cell differentiation. These finding demonstrate that the activation of GSK3β increases cell differentiation (Kuwabara et al., 2009).

Cholinergic neurons are located in extensive regions of the CNS, which regulates complicated behaviors (Hangya et al., 2015). The cholinergic neurotransmission system adjusts the effects of several key factors that are strongly expressed in all cholinergic neurons, termed cholinergic pathway genes (Saunders et al., 2015). Understanding the gene regulatory mechanisms that monitor the expression of cholinergic pathway genes in different groups of cholinergic neurons will provide crucial insights into the process of cholinergic fate specification in CNS diseases (Cho et al., 2014). Previous studies found that seven regulators controlled the identity of cholinergic neuron types. Three LIM homeobox genes (*lim-4/Lhx6/8, lim-11/Lhx1, and ceh-14/Lhx3/4*) and two Prox-type homeobox genes (*unc3/EBF, unc-42/Prd*) control cholinergic identity of cholinergic neuron types, including sensory neurons, interneurons and motor neurons in *Caenorhabditis elegans*. Pitx-type homeobox gene (*unc-30/Pitx)* control the identity of the PVP interneurons (in conjunction with *lin-11*) and POU homeobox gene (*unc-86*/*Brn3*) controls cholinergic identity of the URX, RIH and male-specific CEM neurons (Duerr et al., 2008). lim-7 (*Isl1*), a specific cholinergic identity in the spinal cord and forebrain in the *C. elegans*, has a function as a cholinergic fate determinant in vertebrate CNS (Cho et al., 2014; Zhang et al., 2018).

Methyl 3,4-dihydroxybenzoate (MDHB, C8H8O4), with a molecular weight of 168.15 (CAS), is a small molecular compound extracted from the traditional herbs. Previous researches have described that MDHB has the effect of antioxidant (Cai et al., 2016). In addition, studies in our laboratory have shown that MDHB could accelerate the neurite outgrowth of primary cortical neurons *in vitro* by inducing brain-derived neurotrophic factor (BDNF) expression (Zhang Z. et al., 2015), protect the primary cortical neurons against Aβ(25-35)-induced apoptosis by mitochondria pathway (Zhou et al., 2013), as well as prolong the lifespan of *C. elegans* (Zhang et al., 2014).

In this study, we found that MDHB can specifically induce neuronal differentiation *in vitro* and promote excitatory cholinergic motor neuron differentiation. Additionally, MDHB can increase the activity of tyrosine-phosphorylated GSK3β, and then the activated GSK3β promotes phosphorylation of β-catenin, resulting in the degradation of β-catenin. Subsequently, cell cycle and *Tacc3* gene controlled neuronal differentiation can be inhibited. *Isl1* gene controlled cholinergic neuronal differentiation will be up-regulated. In summary, we showed that the expression of neuronal differentiation transforming acidic coiled-coil 3 (*Tacc3*) gene and cell cycle are inhibited and cholinergic neuronal differentiation gene *Isl1* are up-regulated by MDHB.

## Materials and Methods

### Animals and Ethics Statement

This study was carried out in accordance with the recommendations of the Animal Research Committee of the School of Medicine of Jinan University (Approval Number: 20170607002). The protocol was approved by the Animal Research Committee of the School of Medicine of Jinan University.

### Isolation and *in vitro* Culture of NSCs

Rat NSCs were derived and cultured as described previously by others (Rietze and Reynolds, 2006). Briefly, the hippocampi of several postnatal rats were chopped, mechanical digested by 0.25% trypsin (Gibco) in a humidified 5% CO_2_ incubator at 37°C for 10 min and triturated. The cell suspension was added into an equal volume of DMEM/F12 (Gibco) supplemented with 10%fetal bovine serum (Lonsera) and 0.1 mg/ml DNase I (Sigma), afterward filtered through a 70 μm microfiltration membrane and centrifuged for 5 min. The cells cultured in DMEM/F12 containing 10 ng/mL basic fibroblast growth factor (Proteintech), 20 ng/mL EGF (Proteintech), 1%penicillin and streptomycin (Sigma) and 2% B27 (Gibco) without vitamin A were seeded in 6 well plate in a humidified 5% CO2 incubator at 37°C. Within 3–5 days, the cells grew into free floating neurospheres which were then gathered by centrifugation and passaged after mechanical, dissociation by pipetting.

### NSCs Differentiation

For NSCs differentiation, neurospheres (passage 2–3) were dissociated into a NSC by stem cell digestive enzyme (Gibco) and NSCs were seeded in 0.0125 mg/ml poly-D-lysine (PDL, Sigma) and 10 ng/ml laminin (Sigma)-coated glass cover slips at the density of 35,000 cells/cm^2^ directly in DMEM/F12 supplemented with 1%FBS (Gibco) and 1%penicillin and streptomycin. When cells were completely adherent in the plate after 2 h, DMEM/F12 containing 1%FBS was replaced by rat NSCs differentiation medium (NeuroCult Differentiation Kit, Catalog #05700). The cultures were then treated with MDHB (0, 8, 16, and 32 μM) which was dissolved in DMSO (Sigma). The culture treated with MDHB was changed every second day.

### Immunofluorescence Staining

Treated cells were fixed with 4% paraformaldehyde (PFA) for 45 min at room temperature, washed with phosphate-buffered saline (PBS, pH7.6) and blocked with super blocking solution containing 0.5% goat serum, 1%fish serum, 0.5% donkey serum and 0.5% bovine serum in 0.3% Triton X-100 PBS at room temperature for 60 min. Subsequently cells were incubated with primary antibodies at 4°C for 16 h overnight. The primary antibodies were mouse anti-Nestin (1:100, Millipore), mouse anti-neuron-specific class III beta-tubulin (Tuj-1, 1:1000, Sigma), mouse anti-microtubule-associated protein 2 (MAP2, 1:500, Sigma), and rabbit anti-glial fibrillary acidic protein (GFAP, 1:1000, Abcam), rabbit anti-PSD95 (1:500, Abcam), Mouse anti-CAMKII (1:500, Abcam), rabbit anti-Ki67 (1:500, Abcam), mouse anti-ChAT (1:500, Sigma), mouse anti- VGluT1 (1:500, Millipore), mouse anti-TPH (1:500, Millipore), rabbit anti-Gad67 (1:500, Sigma), rabbit anti-TH (Millipore), goat anti-Isl1 (1:500, Abcam), chicken anti-MAP2 (1:2000, Sigma), rabbit anti-Tbr1 (1:500, Sigma), rabbit anti-Prox1(1:500, Abcam), rat anti-Ctip2 (1:500, Millipore), mouse anti-Cux1(1:500, Sigma), rabbit anti-NeuN (1:1000, Abcam). The cells were washed three times with 0.3% PBST and incubated with Alexa Fluor 488, CY3, 647-conjugated secondary antibody (1:1000, Earthox) at room temperature for1h. To visualize nuclei, cells were counterstained with 1 ng/ml 4′,6-diamidino-2-phenylindole (DAPI, 1 ng/ml, Sigma) for 5 min. Finally, all images were captured with a confocal microscope (Zeiss, LSM700) and then processed via Image J software (NIH, Bethesda, MD, United States). The number of Nestin, Tuj-1, MAP2, GFAP, Nestin, ChAT, NeuN, Cux1, Ctip2, Tbr1 and Prox1 positive cells and cell nuclei were counted in each of seven random fields per well.

### Western Blot Analysis

Cells differentiate for 5 days in differentiation medium in the presence of MDHB and then collected. Cells were washed with pre-cooling phosphate-buffered saline (pH7.6) added with the lysis buffer, and then they were homogenized via ultrasonication and centrifuged at 12000 *g* for 10 min at 4°C. The protein concentration in the supernatant was detected by a BCA assay kit (Beyotime Institute of Biotechnology). Then the supernatants were blended with loading buffer in a ratio of 1:1 and boiled for 5 min at 100°C, and then subjected to SDS page (12% and 10% acrylamide gels, 120 V, 1.5 h). The separated proteins were transferred to poly-vinyli dene fluoride (PVDF) membranes (100 V, 85 min) and blocked with 5% skimmed milk dissolved in 0.05%TBST. After three times rinsing in 0.05%TBST, the proteins were incubated with primary antibodies overnight at 4°C. The membrane was exposed to either HRP- rabbit or mouse secondary antibody for 1 h at room temperature. The fluorescent signal of the blots was collected by ALLIANCE 4.7 apparatus and quantified with the Quantity One software. The expressions of β-III-tubulin, GFAP, AKT (CST), p-AKT (CST), GSK3β (CST), p-ser9-GSK3β (CST), p-try-GSK3β (Thermofisher) and β-catenin (CST) were determined via calculating their density ratio to the GAPDH band.

### Cell Collection and mRNA Preparation

Cells were collected after differentiating for 5d in differentiation medium in the presence of MDHB. Total RNA was isolated using Trizol Reagent (Invitrogen), in combination with RNAase-free DNAase to eliminate the potential DNA contamination (TAKARA). The concentration and purity of RNA were measured by Nanodrop 2000C Spectrophotometer.

### Transcriptome Analysis of MDHB-Induced Differentiation

The RNA-seq technique was used to analyze the gene expression profiling in MDHB-induced NSCs differentiation (SRA accession:PRJNA505930). The cDNA fragments were purified using a QIAquick PCR extraction kit following the manufacturer’s protocol. Next, the cDNA fragments were enriched by PCR to construct the final cDNA, which was sequenced by Illumina sequencing platform (IlluminaHiSeq^TM^ 2500).

### Gene Expression Validation by qRT-PCR

Reverse transcription was performed into cDNA using the PrimeScript TMRT reagent Kit with gDNA Eraser following the manufacturer’s protocol. Real-time PCR was performed with a SYBR^®^ Premix Ex TaqTM II detection System. The primer sequences are shown as follows (Table 1):

**Table 1:** The primer sequences.

Gene	Primer sequence
Cdkn1a-Forward	5-GGGATGCATCTATCTTGTGATATG-3
Cdkn1a-Reverse	5-GCGAAGTCAAAGTTCCACCG-3
Cdc20-Forward	5-TGGAGAAAGTGGCTGGGTTC-3
Cdc20-Reverse	5-ATGCGAATGTGTCGGTCACT-3
Tacc3-Forward	5-GTCTGGCTCCGGAAATCCAA-3
Tacc3-Reverse	5-CACCATAGGCTCGGCAGGAA-3
Actin-Forward	5-CACCCGCGAGTACAACCTTC-3
Actin-Reverse	5-CCCATACCCACCATCACACC-3
Isl1-Forward	5-GACATGATGGTGGTTTACAGGC-3
Isl1-Reverse	5-GCTGTTGGGTGTATCTGGGAG-3
Lhx8-Forward	5-CAGTTCGCTCAGGACAACAA-3
Lhx8-Reverse	5-AGCCATTTCTTCCAACATGG-3
Lhx3-Forward	5-AGAGCGCCTACAACACTTCG-3
Lhx3-Reverse	5-GGCCAGCGTCTTTCTTCAGT-3

### Statistical Analysis

The data are expressed as mean ± SEM. Statistical analyses were performed by using a Test or ANOVA followed by Boferonni’s test, using the Prism software (GraphPad, San Diego, CA, United States). A value of *P* < 0.05 was considered as significantly different from the control.

## Results

### Neural Stem Cells, Culture and Identification

**FIGURE 1:**
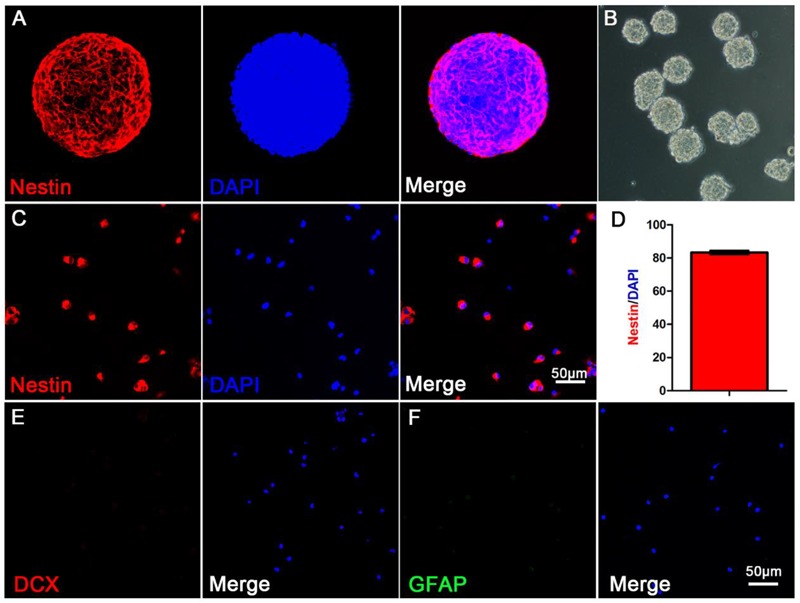
The morphological and purity identification of primary neurospheres and neural stem cells. **(A)** Nestin and DAPI fluorescent staining of primary neurospheres composed of neural stem cells; **(B)** The morphological status of primary neurospheres *in vitro*; **(C)** Nestin and DAPI fluorescent staining of scattered primary NSCs; **(D)** The purity statistics of primary NSCs; **(E)** DCX and DAPI staining of scattered primary NSCs; **(F)** GFAP and DAPI staining of scattered primary NSCs.

To extract primary hippocampal NSCs, neurospheres were passaged *in vitro* (Reynolds and Rietze, 2005). Experimental materials were prepared from the second or the third generation of NSCs. The third generation of NSCs (Figure 1B) were most suspended neurospheres. Nestin and DAPI were used to stain single neural stem cell (Figure 1C) from digested neurospheres by immunofluorescence (Figure 1A). Analysis showed that the purity of the primary cells were most NSCs (Figure 1D), and DCX (neurons marker) and GFAP (astrocytes marker) were immunonegative of primary NSCs (Figures 1E,F).

### MDHB Promotes the Differentiation of NSCs Into Neurons

**FIGURE 2:**
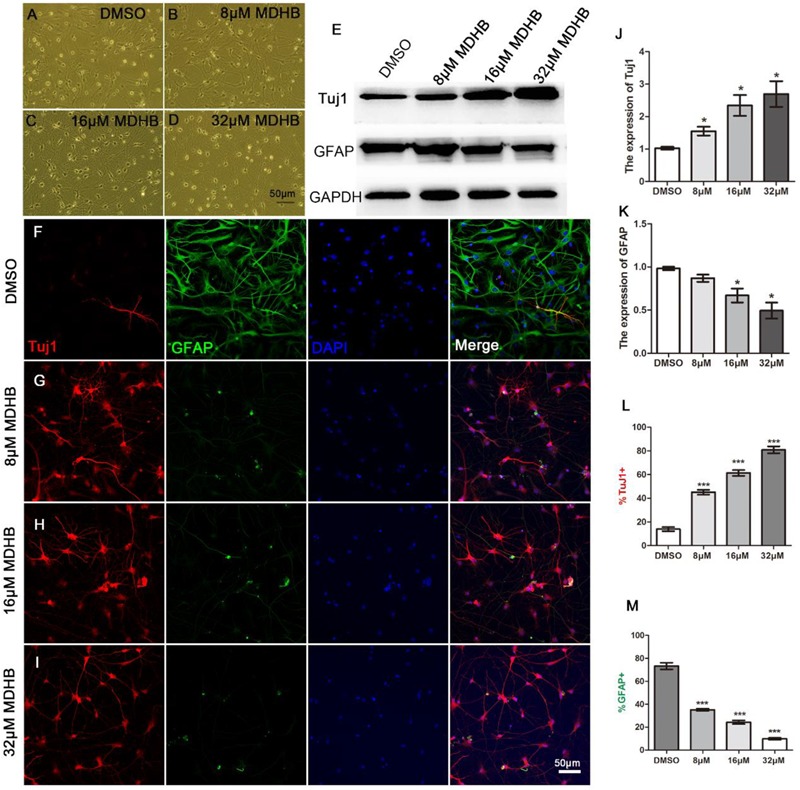
MDHB promotes NSCs differentiation into neurons. **(A)** The solvent control group (DMSO); **(B–D)** Low dose (8 μM MDHB), middle dose (16 μM MDHB), and high dose (32 μM MDHB) groups. **(E)** Western blot analysis for Tuj1 (immature neuron) and GFAP (astrocyte) on differentiation of NSCs. **(F–I)** After 5 days of NSCs differentiation induced by MDHB, new cells were fluorescently stained, the cells with neuronal marker Tuj1 were dyed red; with glial marker GFAP dyed green; and all cell nuclei with nuclear marker DAPI were dyed blue. **(J,K)** Quantification of protein blots is shown, GAPDH serves as protein loading control. **(L)** The statistical results of neurons differentiated by MDHB-induced NSCs. **(M)** The statistical results of astrocytes differentiated by MDHB-induced NSCs (^∗^*P* < 0.05, *^∗∗∗^P* < 0.001, compared with DMSO group, *n* = 3).

To determine the effects of different concentrations of MDHB on neuronal differentiation of NSCs, neurospheres were dissociated into a single NSC, which was treated in the presence of the following concentrations: 0 μM MDHB, 8 μM MDHB, 16 μM MDHB, 32 μMMDHB. After 5 days, we observed that the cell bodies of different concentrations of MDHB groups had neuronal morphological features (Figures 2A–D). The expressions of Tuj1 (immature neuron) and GFAP (astrocyte) were detected by western blot (Figures 2E,J,K). The results revealed that MDHB increased the expression of Tuj1 and inhibited the expression of GFAP in the differentiation of NSCs. Neuronal marker Tuj1 and astrocyte marker GFAP were used to identify cells (Ray et al., 2018) (Figures 2F–I). The DMSO group had more GFAP positive cells, while MDHB treated group (8, 16, and 32 μM) had more Tuj1 positive cells. Above results indicated that MDHB enhanced the differentiation of NSCs into neurons (Figure 2J) and inhibited NSCs differentiation into astrocytes (Figure 2K).

### MDHB-Induced Immature Neurons Form Mature Neurons

**FIGURE 3:**
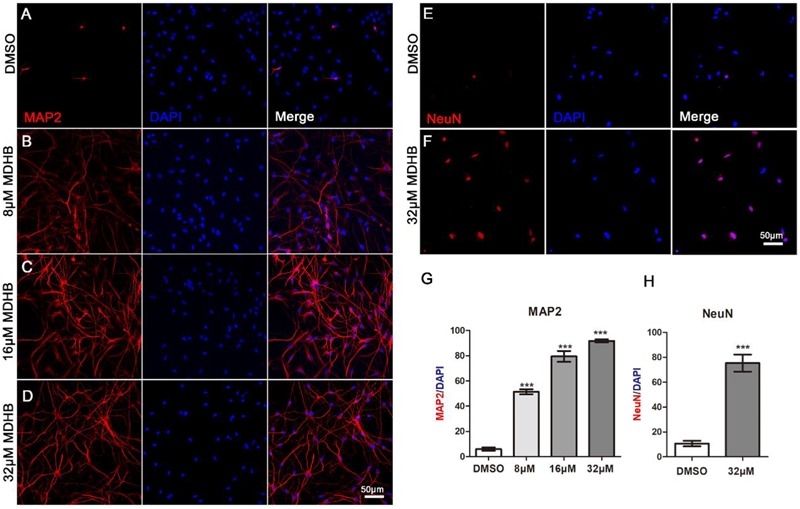
MDHB-induced immature neurons form mature neurons. **(A–F)** After 9 days of NSCs differentiation induced by MDHB, the new cells were fluorescently stained, the cells with MAP2 and NeuN were dyed red and the cell nuclei with DAPI was dyed blue; **(G,H)** the statistical results of mature neurons differentiated by MDHB-induced NSCs (*^∗∗∗^P* < 0.001, compared with DMSO group, *n* = 3).

Next, we examined whether immature neurons in MDHB-induced differentiation can form mature neurons. After 9 days using different doses of MDHB presenting on NSCs, cells were stained with MAP2 and NeuN marker which are specific markers of mature neurons (Figures 3A–F). The result showed that MAP2 positive cells were significantly increased in a MDHB dose-dependent way, and NeuN positive cells were also promoted by MDHB-induced NSCs differentiation. Thus, MDHB can promote NSCs differentiation into mature neurons (Figures 3G,H).

### MDHB Promotes the Differentiation of NSCs Into Cholinergic Neurons

**FIGURE 4:**
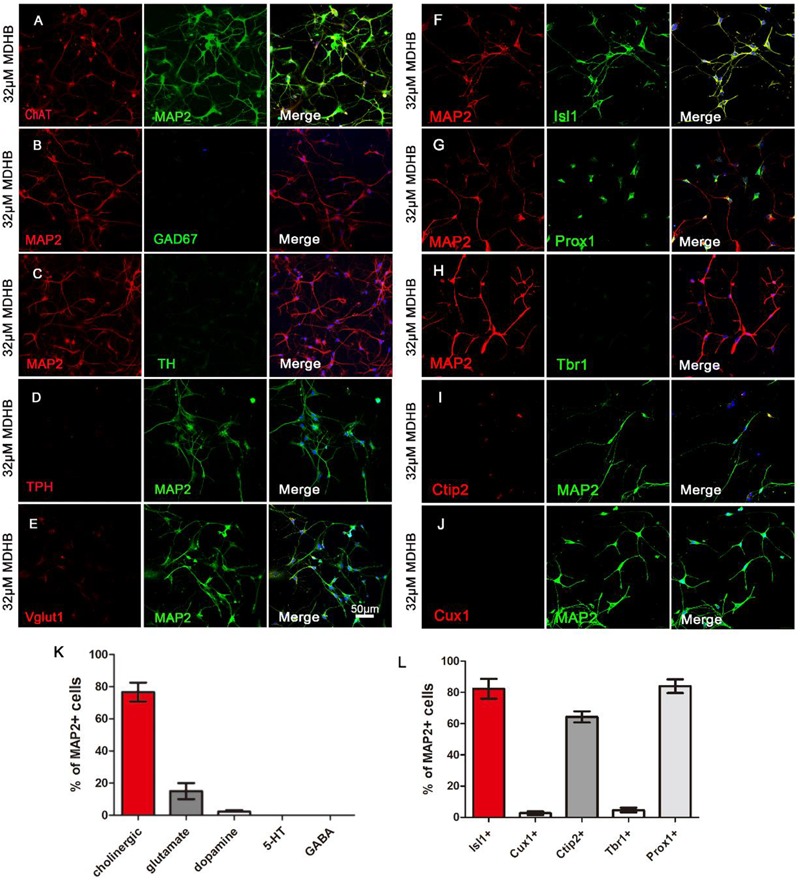
The types analysis of MDHB-induced neurons. **(A)** The cholinergic neurons with ChAT and MAP2 were respectively dyed red and green; **(B)** the GABAergic neurons with MAP2 and Gad67 were respectively dyed red and green; **(C)** the dopaminergic neurons with MAP2 and TH were respectively dyed red and green; **(D)** the serotonergic (5-HT) neurons with TPH and MAP2 were respectively dyed red and green; **(E)** the glutamatergic neurons with Vglut1 and MAP2 were respectively dyed red and green; **(F)** MDHB-induced neurons were also immunopositive for motor neuron marker Isl1; **(G,H)** Immunostaining with neuron markers revealed that MDHB-induced neurons were negative for general cortical neuron marker Tbr1, but positive for hippocampal neuron marker Prox1 **(G)**; **(I,J)** Immunostaining with neuron markers revealed that MDHB-induced neurons were negative for superficial layer marker Cux1 **(J)**, but positive for deep layer marker Ctip2 **(I)**. **(K,L)** The statistical results showed that MDHB promoted NSCs differentiation into hippocampal cholinergic neurons.

Here, we investigated neuronal subtypes based on neurotransmitters they contain. We first found that the majority of MDHB-induced neurons were immunopositive for cholinergic neurons marker ChAT (Figure 4A) and motor neurons marker Isl1 (Figure 4F). A small fraction of the converted neurons was immunopositive for glutamatergic neurons marker VGluT1 (Figure 4E). On the other hand, the MDHB-induced neurons were immunonegative for GABAergic neurons marker GAD67 (Figure 4B), dopaminergic neurons marker TH (Figure 4C), and serotonergic (5-HT) neurons marker TPH (Figure 4D). The quantitative analyses of the neuronal subtypes were shown in Figure 4F (*n* = 3 batches). These results suggested that the cholinergic motor neurons were the major subtype of MDHB-induced neurons.

To characterize the neuronal properties after small molecule-induced differentiation, we examined neuronal markers expressed from anterior to posterior nervous system. We found that the MDHB-induced neurons were- immunonegative for superficial layer neuronal marker Cux1 (Figure 4J), but positive for deep layer neuronal markers Ctip2 (Figure 4I). The MDHB-induced neurons were also immunonegative for cortical neuronal marker Tbr1 (Figure 4H), as well as hippocampal neuronal marker Prox1 (Figure 4G). Figures 4K,L showed the quantitative results. Therefore, our MDHB-induced neurons were mainly hippocampal neurons (Figures 4K,L).

### The Expression of Synaptic Proteins in MDHB-Induced Neurons

**FIGURE 5:**
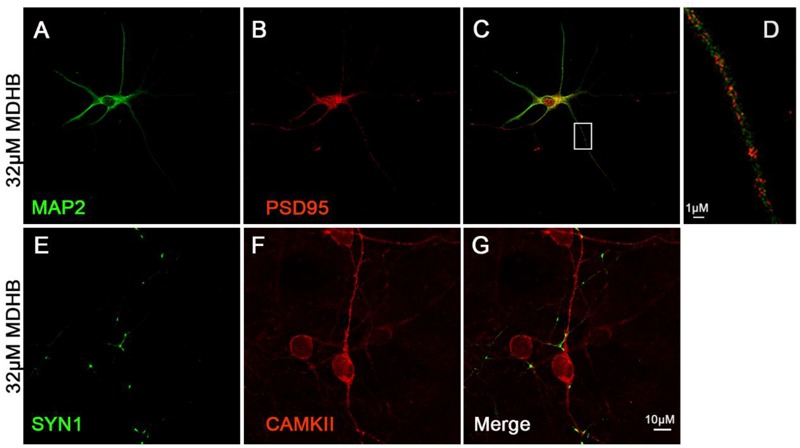
The expression of synaptic protein in MDHB –induced neurons. **(A–D)** MDHB –induced neurons has the expression of postsynaptic dense protein (PSD95); **(E–G)** MDHB –induced neurons has the expression of Synapsin I (SYN1).

We next investigated whether MDHB-induced neurons have synapse formation. Synapsin I exists in the nerve terminal of axons, mainly in the membranes of synaptic vesicles. The encoded protein acts as a substrate for several different protein kinases, and phosphorylation can play a role in the regulation of proteins in the nerve terminal. PSD-95 is a member of the membrane-associated guanylate kinase (MAGUK) family, and it plays an important role in synaptic plasticity and the stabilization of synaptic changes (Perche et al., 2018; Whalley, 2018). Calcium/calmodulin-dependent protein kinase II (CaMKII)-the main protein of the postsynaptic density-is a Ca^2+^/calmodulin-activated dodecameric enzyme (Lisman et al., 2002). The expression of synaptic proteins in neurons was identified by cellular immunofluorescence. The Figure showed that Synapsin I (SYN1) (Figures 5E,F) and postsynaptic density protein 95 (PSD-95) (Figures 5A–D) significantly expressed. It indicated that these neurons could form neural network.

### Effect of MDHB on AKT, GSK3β and β-catenin

**FIGURE 6:**
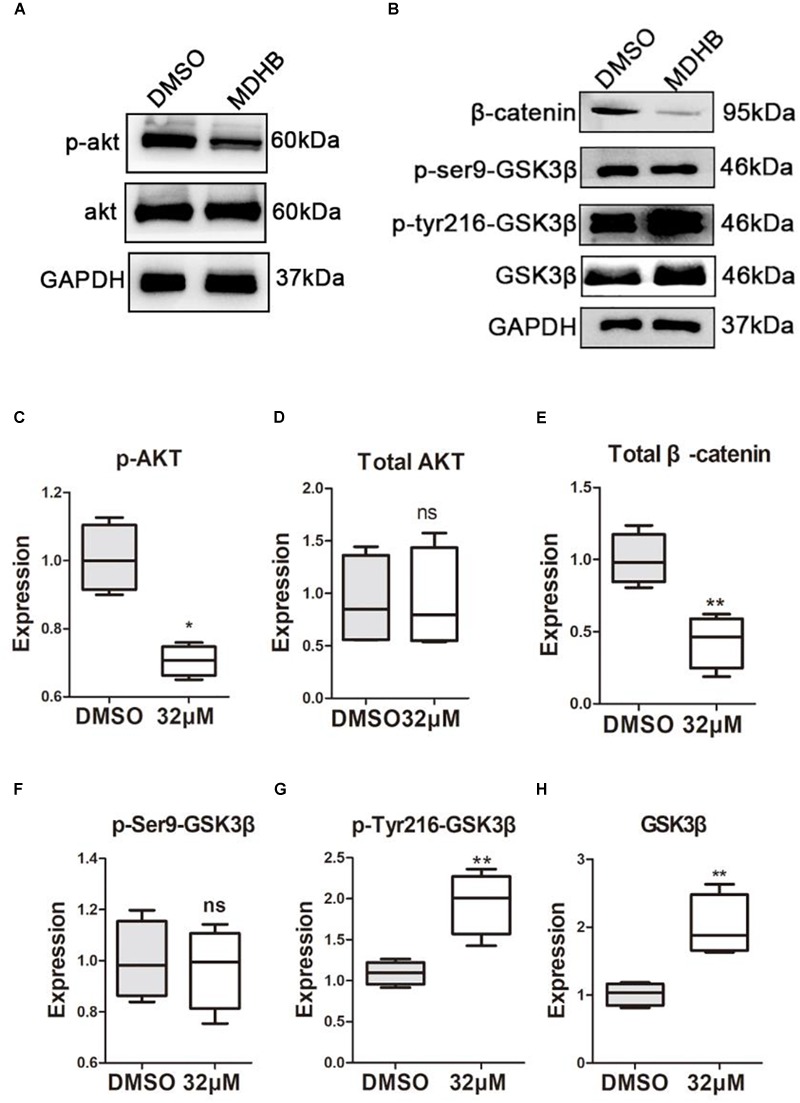
The effect of MDHB on AKT, GSK3β and β-catenin. MDHB inhibited phosphorylation of AKT in the differentiation of neural stem cells, activated phosphorylation of GSK3β at tyrosine 216 (Y216), and downregulated transcription factor β-catenin. **(A,B)** Western blot analyses of proteins extracted from MDHB-treated neural stem cell in differentiation, **(C–H)** quantification of protein blots is shown, GAPDH serves as protein loading control. Each point represents the mean relative protein level of each group (*^∗^P* < 0.05, compared with DMSO group, *^∗∗^P* < 0.01, compared with DMSO group, *n* = 4).

To understand the molecular mechanisms of MDHB-induced differentiation of NSCs into cholinergic neurons (Figures 6A,B). Western blot results revealed that the phosphorylation level of AKT protein was down-regulated in the MDHB group (Figure 6C), and the total AKT protein expression was unchanged (Figure 6D), MDHB activated phosphorylation of GSK3β at tyrosine 216 (Y216) (Figure 6F). The phosphorylation level of serine at position 9 was unchanged (Figure 6G) and the transcription factor β-catenin was down-regulated (Figure 6E). As shown above, the mechanism of MDHB promoted differentiation of NSCs into cholinergic neurons may perform by increasing. The activity of GSK3β (Figure 6H) and inhibiting the activity of PI3K.

### MDHB Inhibits Cell Cycle and Increases the Expression of Cholinergic Neuronal Gene *Isl1*

**FIGURE 7:**
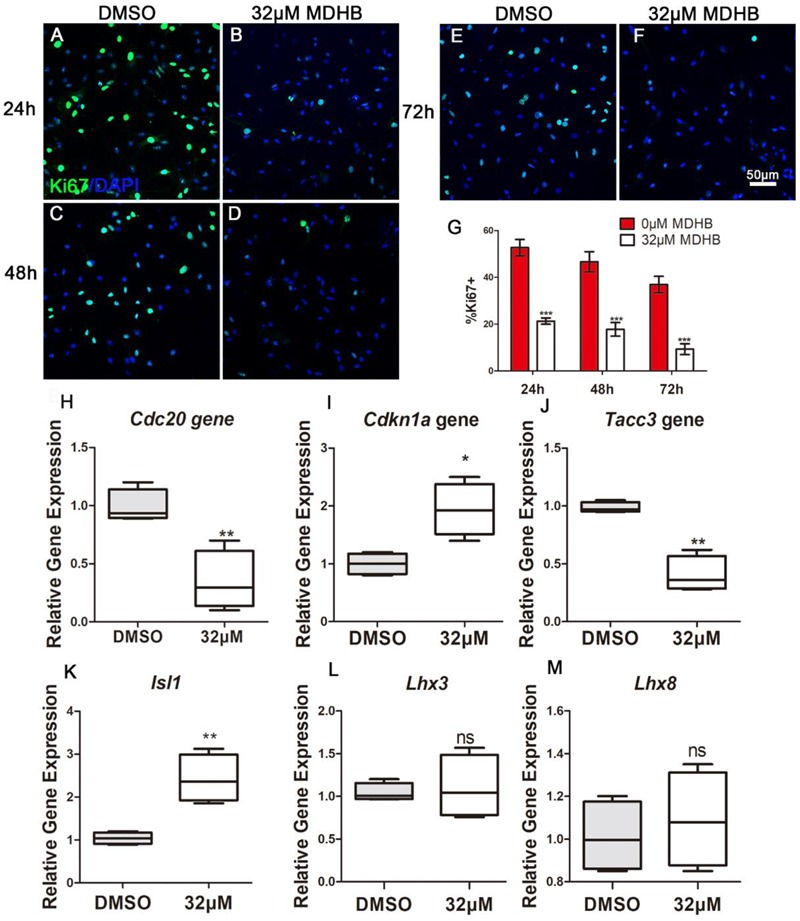
MDHB promotes the differentiation of NSCs into cholinergic neurons by inhibiting cell cycle and increasing cholinergic pathway gene *Isl1*. **(A)** The effect of DMSO on differentiation of NSCs for 24 h; **(B)** the effect of MDHB on differentiation of NSCs for 24 h; **(C)** the effect of DMSO on differentiation of NSCs for 48 h; **(D)** The effect of MDHB on differentiation of NSCs for 48 h; **(E)** the effect of DMSO on differentiation of NSCs for 72 h; **(F)** The effect of MDHB on differentiation of NSCs for 72 h; **(G)** the statistical results of Ki67 staining for different time point. **(H)** The relative expression levels of *Tacc3* gene; **(I)** the relative expression levels of *Cdc20* gene; **(J)** the relative expression levels of *Cdkn1a* gene; **(K)** the relative expression levels of *Isl1* gene; **(L)** the relative expression levels of *Lhx3* gene; **(M)** the relative expression levels of *Lhx8* gene (*^∗^P* < 0.05, compared with DMSO group; *^∗∗^P* < 0.01, compared with DMSO group; *^∗∗∗^P* < 0.001 compared with DMSO group, green = ki67, blue = DAPI).

The transcription factor β-catenin was down-regulated, which is critical for the control of NSCs’ cell cycle in multiple regions of the developing CNS. We further performed immunostaining to examine the cell cycle protein expression changes during induced NSCs differentiation process. MDHB was utilized to treat NSCs in 24, 48, and 72 h for differentiation. The data showed that the expression of Ki67 in MDHB group was less in 24, 48, and 72 h (Figures 7A–G). Compared with the DMSO group, the expression of *Tacc3 in* neuronal differentiation and *Cdc20* in cell cycle were decreased, and the expression of *Cdkn1a* in cell cycle and *Isl1* in cholinergic pathway was up-regulated. *Lhx3* and *Lhx8* had not change. Our data indicated that MDHB induced NSCs differentiation by regulating cell cycle-related gene and cholinergic neuron-related gene.

## Discussion

Since the neuronal differentiation of NSCs is an intricate process, it is not surprising that many substances have been involved in the regulation action (Gauthier-Fisher et al., 2009; Ming and Song, 2009; Akizu et al., 2010; Luo et al., 2010; Park et al., 2016; Vasconcelos et al., 2016). However, little is known about the small molecules in NSCs. The small molecules controlling the direction of NSCs differentiation would be a critical advance in neurodegeneration and CNS repair. Here, we described a novel small molecule -named MDHB that modulates cholinergic neuronal differentiation of NSCs. We found that MDHB promotes the differentiation of NSCs into neurons and inhibits NSCs differentiation into astrocytes. The major subtypes of MDHB-induced neurons are cholinergic neurons.

NSCs refer to a type of cell population that exists in CNS and has the latent energy to differentiate into neurons, astrocytes, and oligodendrocytes, and they can proliferate to replenish lost brain cells (Mosher et al., 2012; Aharonowiz et al., 2018). Mouse NSCs were successfully isolated and established in 1992. Subsequently, NSC lines of human and other organisms were successively established(Kim et al., 2008; Chicheportiche et al., 2018). We performed Nestin and DAPI fluorescent staining of NSCs, which is widely used as a specific marker of NSCs (Crook and Tomaskovic-Crook, 2017; Jin et al., 2017). The results demonstrated that the high purity of NSCs in the cultured primary cells.

These results indicated that MDHB (8, 16, and 32 μM) promotes NSCs differentiation into immature neurons and overrides NSCs differentiation into astrocytes. In this study, the effect of MDHB on the differentiation of NSCs was observed. The whole cell exhibited obviously neuronal morphological characteristics. Tuj1 is a neuron-specific marker which has been widely used for the identification of immature neurons, GFAP is an astrocytes marker which is generally used for identification of glial cells(Park et al., 2017). These results indicated that MDHB (8, 16, and 32 μM) promotes differentiation of NSCs into immature neurons while inhibiting their differentiation into astrocytes (Figure 2). MAP2 is the skeleton protein of neurons and plays a vital role in the stability and function in maintaining microtubules. It widely distributes in mature neuronal dendrites and is used as a marker to measure the growth of neuronal processes (Gumy et al., 2017). NeuN is also a mature neuronal marker in the neuronal nucleus. Through mature neuronal immunofluorescence staining, we found that the expressions of MAP2 and NeuN in MDHB groups are significantly higher than the DMSO group in a dose-dependent manner. It indicated that MDHB can promote not only NSCs differentiation into immature neurons, but the development into mature neurons.

Neurons have a wide variety of classifications, which include basis on the structure of neurons, the function of neurons and neurotransmitters releasing (Zeng and Sanes, 2017). At present, according to the neurotransmitters, they are classified into the following categories: cholinergic neurons, dopaminergic neurons, GABAergic neurons, serotonergic (5-HT) neurons, glutamatergic neurons and so on (Dvoryanchikov et al., 2017). Neurons released different neurotransmitters have distinct functions in the CNS (Haim and Rowitch, 2017; Zhou et al., 2017). According to the localization that they place in brain, they can be classified into superficial layer neurons, deep layer neurons and so on (Zeng and Sanes, 2017). Previous research showed that the main hippocampal neurons were Glutamatergic and GABAergic (Lei et al., 2016). Although the cholinergic neuron is not the main type in the hippocampus, they still play a major function in the hippocampus. Our results demonstrated that ChAT was the marker of cholinergic neurons, while Prox1 and Ctip2 were the markers of hippocampal and deep layer neurons respectively (Zhang L. et al., 2015). Recent study showed that single cholinergic neuron can extend into multiple areas. Most target areas of individual cholinergic neuron are interconnected, such as the olfactory bulb connecting to the piriform and entorhinal cortex, while the subregions of the hippocampal complex connecting with the striatum, isocortex, and hypothalamus (Li et al., 2018). Interestingly, our research has demonstrated that most of MDHB-induced neurons were cholinergic neurons. But this also prompt that it is precisely because MDHB can differentiate NSCs into cholinergic neurons, thus acting as a therapeutic effect for memory disorder. In the near future, we will need to further verify the function of this part of cholinergic neurons. Due to the potential limitations of our experimental conditions, we were unable to identify the exact type of the differentiated cells using multiple experimental methods, which could not totally exclude the possibility of other cell types. So it is worthy of further research in this direction. A semiautonomous circuit of striatal GABAergic interneurons is responsible for transmitting behaviorally relevant cholinergic signals to spiny projection neurons (Faust et al., 2016). Cholinergic neuron play a major role in motor and learning functions of the striatum. At recent studies, the cholinergic neurons have also been a main focus of research in aging and neural degradation, specifically as it relates to Alzheimer’s Disease (Tabet, 2006). Through the observation and analysis of the staining of presynaptic membrane protein SYN1 and PSD95, the results showed that the differentiated neurons expressed SYN1 and PSD95 (Figure 5), suggesting the possibility of synapse formation and the potential to form neural networks. The experiments revealed that MDHB can promote NSCs differentiation into hippocampal cholinergic neurons, and the differentiated neurons may form a neural network.

Previous research showed that Wnt pathway promotes neuronal differentiation (Hirabayashi et al., 2004). In the classical WNT pathway, inhibition of GSK3βleads to the accumulation of β-catenin, which enters the cell nucleus, causing transcription of the *TCF4* gene (Hirabayashi et al., 2004). GSK3β is also regulated by the PI3K/AKT signaling pathway. Activated by AKT (Jiang et al., 2016), inhibits GSK3β activity, In contrast, inactivating AKT, activates GSK3β activity. PI3K/AKT signaling pathway regulate NSCs differentiation into motor neurons in adult. In this study, we found that MDHB regulates the fate of NSCs by regulating cell cycle, possibly inhibiting AKT phosphorylation and activating GSK3β activity, which lead to β-catenin degradation and abolishment of entering the nucleus. Subsequently, it will regulate cell cycle-related gene expression and cholinergic signal pathway. Recent studies have found that transforming coiled coil acid repeat protein 3 (TACC3) can regulate ARNT2 transcription factor to determine neural cell fate (Wurdak et al., 2010). Knocking out the *TACC3* gene can promote the differentiation of NSCs into neurons (Xie et al., 2007). Cell division cyclin 20 is a basic regulator of cell division, and it have an important function to activate late-promotion complex (APC/C), which is a large 11-13 subunit complex that initiates staining (Paul et al., 2017; Omrani et al., 2018). The body separates and enters later stages, it also targets the destruction of S and M phase (S/M) cyclins, inactivates S/M cyclin-dependent kinases (Cdks) and quits cells from mitosis (Lee et al., 2018). The LIM homeodomain transcription factor Islet1 (*Isl1*) is expressed in multiple organs and plays essential roles during embryogenesis. Isl1 is required for the survival and specification of motor neurons, *Isl1* orchestrates the process to generate cholinergic neurons in the spinal cord and forebrain. In this experiment, the expressions of *Cdc20* and *TACC3* are decreased and the expressions of *Cdkn1a* and *Isl1*gene are up-regulated after NSCs was exposed to MDHB (Figure 7).

**FIGURE 8:**
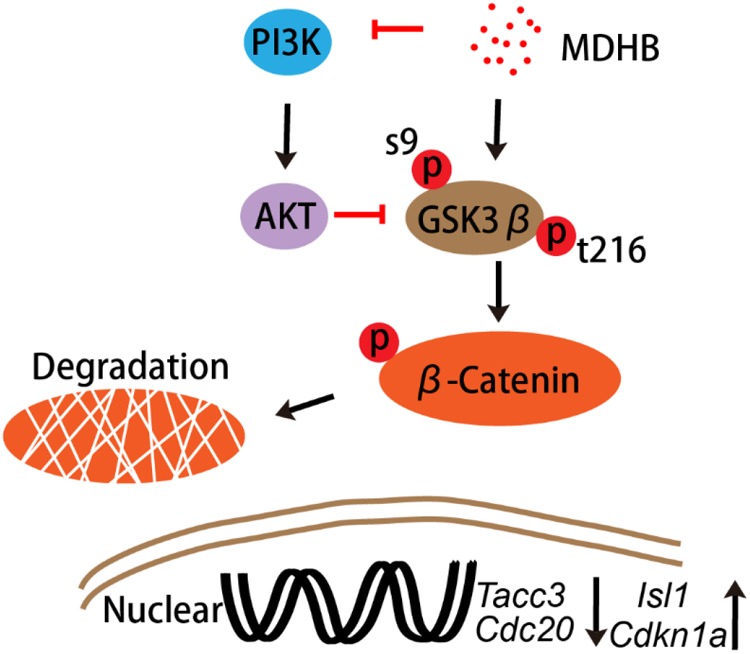
The signal transduction pathway in MDHB-induced cholinergic neurons differentiation.

In this study, we showed that MDHB may induce NSCs to differentiate into cholinergic neurons by regulating cell cycle-associated proteins and cholinergic signal pathway (Supplementary Figure S1). Ki67 is a nuclear protein associated with cellular proliferation (Calzolari et al., 2015). The results were consistent with the transcriptome levels. Western blot was used to detect the proteins which included the phosphorylation of AKT and total AKT in the PI3K pathway at the fifth day of NSCs differentiation. We found that MDHB inhibited the phosphorylation of AKT, but the total amount of AKT was not affected. Subsequently, the classical WNT pathway protein was verified, and the data that MDHB regulates phosphorylation of GSK3β at tyrosine 216 (Y216), but does not cause phosphorylation of GSK3β at serine 9 (S9). MDHB activates GSK3β and cause β-catenin degradation which leads to the inability to enter the nucleus and initiate the expression of cholinergic-related genes and cell cycle-related genes. At the transcriptome level, it was also found that the expressions of *Cdc20* and *Tacc3* were decreased and *Cdkn1a and cholinergic* genes was upregulated. The qPCR assay of *Isl1, Cdc20, Tacc3*, and *Cdkn1a* revealed that *Cdc20* and *Tacc3* were down-regulated, Isl1 expression was also increased by immunofluorescence. In this process, β-catenin may control the expression of the *Isl1* and *Tacc3*. In summary, MDHB may promote NSCs differentiation into cholinergic neurons by inhibiting the proteins in the PI3K signaling pathway and activating the proteins in GSK3β signaling pathway to regulate the expression of cholinergic gene (Figure 8). The differentiation-inducing agents such as MDHB may lead to new therapeutics that act by enhancing the contribution of newborn neurons to neurodegeneration and CNS repair.

## Author Contributions

H-ML conceived and designed the study. J-PP performed the experiments, analyzed the data and wrote the manuscript. J-PP and YH analysis was performed using the OmicShare tools, a free online platform for data analysis (http://www.omicshare.com/tools). YH, FX, LY, and Y-RX assisted in performing the research. LY, J-HW, J-XJ, K-QC, C-YY, and QG provided language help and assisted in analyzing data.

## Conflict of Interest Statement

The authors declare that the research was conducted in the absence of any commercial or financial relationships that could be construed as a potential conflict of interest.
