# Supplementary material for: Methyl 3,4-Dihydroxybenzoate Induces Neural Stem Cells to Differentiate Into Cholinergic Neurons in vitro
Source: Front Cell Neurosci. 2018 Dec 7;12:478. doi: 10.3389/fncel.2018.00478 (PMC6292956; doi:10.3389/fncel.2018.00478)
Supplement: Supplementary file 1 [file Data_Sheet_1.docx]

**Supplement Material**

The scatter diagram results showed differentially expressed genes between 0μM and 32μM MDHB-inducing NSCs differentiation. A total of 1980 genes showed significant differential expression. Among these genes, 953 genes were up-regulated and 1027 genes were down-regulated(**Fig.S1A**). In order to explore the mechanism, we performed KEGG pathway analysis of the dysregulated genes between between 0μM and 32μM MDHB.The results indicated that cell cycle pathway may be involved in MDHB-induced cholinergic neurons. The KEGG resultsof the top 20 pathways enrichment are shown in Fig. S1B. We made further analyze and cholinergic fate（**Fig.S1C-D**）. Our data may thus include other-related genes not listed among the assigned cholinergic fate genes and provide a useful reference resource for future studies. Functional interaction network analysis revealed that 57 genes belong to the cell cycle and cholinergic function interaction network (**Fig.S1E**), which wasconsistent with the induction of neurons differentiation observed in the NSCs.


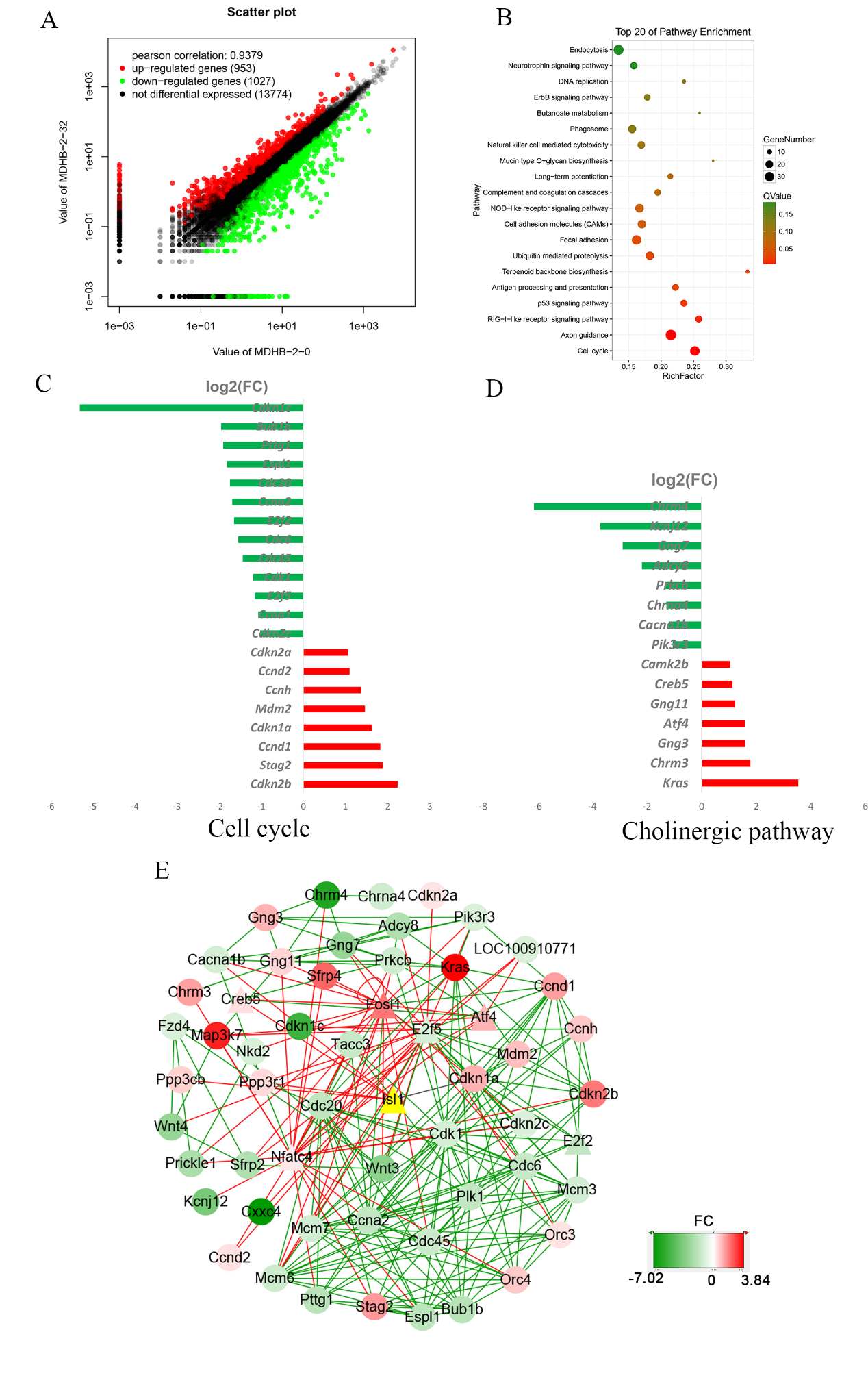


**Figure.S1 Pathways and genes differentially regulates MDHB-induced differentiation in NSCs. A:** Comparison of Scatter plot of between 0μM MDHB and 32μM MDHB; **B:** Comparison of KEGG enrichment of pathway; **C:** Unigenes involved in cell cycle pathways; **D:**Unigenes involved in cholinergic pathways. **E:** A simplified scheme showing the functional interaction network between cell cycle and cholinergic pathway involved in MDHB-induced cholinergic neuron . A total of 57 differentiation-related, differentially expressed genes were applied to Cytoscape analysis (version 2.8.2) installed with Reactome FI plugin.
